# Genome Sequences of Gordonia rubripertincta Phages LilyPad and PokyPuppy

**DOI:** 10.1128/mra.00958-22

**Published:** 2022-10-31

**Authors:** Ella Eleven, Casia Esparza, Alivia Abernathy, Ashley Bradshaw, Marisa Garcia, Nathaniel Jobe, Kennedi Pyper, Cassandra Skaar, Kaarin Goncz, Joel Sharbrough, Linda C. DeVeaux

**Affiliations:** a Biology Department, New Mexico Institute of Mining and Technology, Socorro, New Mexico, USA; Portland State University

## Abstract

Two lytic phages infecting Gordonia rubripertincta were isolated from irrigated desert soil. Phage LilyPad and PokyPuppy have 64,158-bp and 77,065-bp genomes, respectively. Based on gene content similarity to phages in the Actinobacteriophage database, LilyPad is assigned to phage subcluster DG1 and PokyPuppy to subcluster CS2.

## ANNOUNCEMENT

*Gordonia* members are Gram-positive aerobic heterotrophs, of which some can degrade styrene (1). Bacteriophages that infect *Gordonia* may therefore be important in the exchange of bioremediation genes (2, 3). Here, we describe the characteristics of two lytic bacteriophages that infect Gordonia rubripertincta.

**FIG 1:**
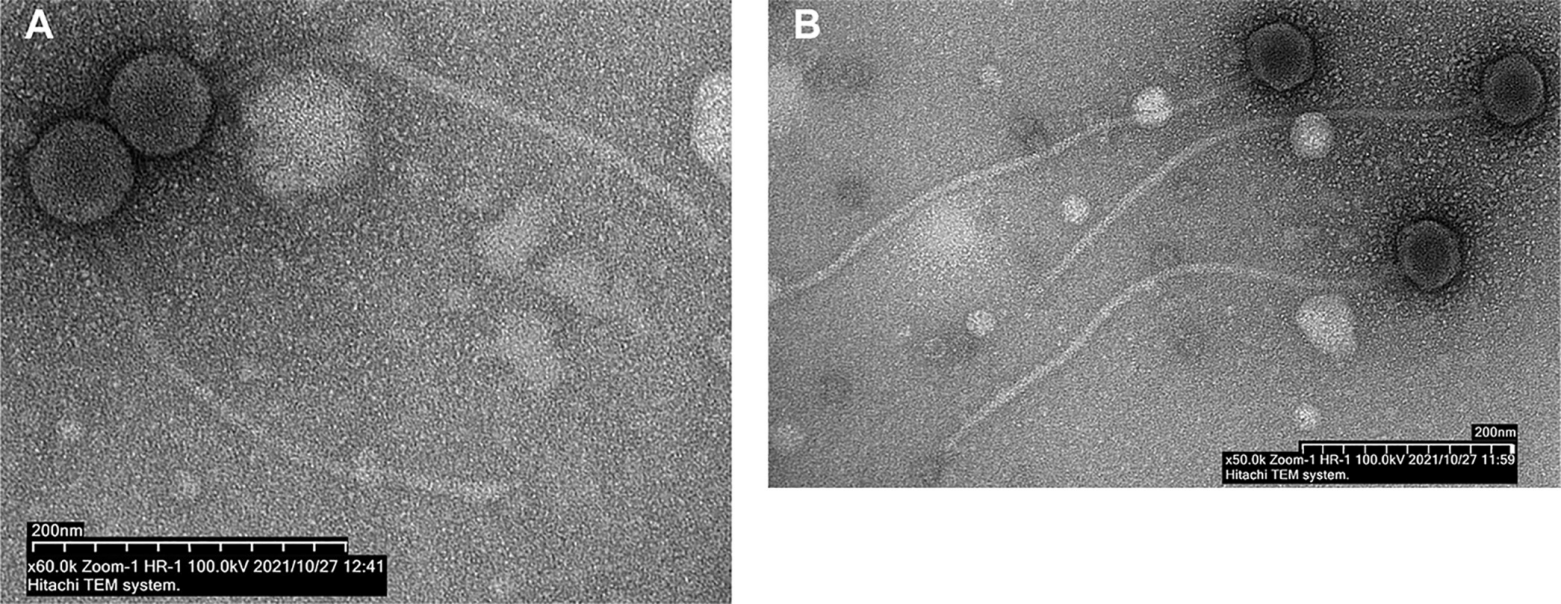
Micrographs of LilyPad and PokyPuppy virions. (A and B) Transmission electron micrographs depicting LilyPad (A) and PokyPuppy (B). LilyPad has an isometric head with a diameter of 62 to 69 nm and a flexible tail 315 to 336 nm in length (n = 6); PokyPuppy has a hexagonal head 58 to 69 nm in diameter and a long flexible tail 465 to 520 nm in length (n = 10).

Phages LilyPad and PokyPuppy were isolated from irrigated soil near the New Mexico Institute of Mining and Technology in Socorro, New Mexico (Table 1), using standard methods (4). Soil samples were resuspended in peptone-yeast extract-calcium medium, and the suspension was spun and filtered (0.22-μm pore size). LilyPad, which was isolated directly from the filtrate by plating in top agar with *G. rubripertincta*, forms clear ~1-mm plaques. PokyPuppy was isolated following inoculation of the filtrate with *G. rubripertincta*, incubation for 3 days at 30°C, and refiltration before plating in top agar with *G. rubripertincta*. PokyPuppy forms ~0.1-mm plaques. Both phages were purified by plating 2 to 3 times and form plaques after 2 days at 30°C. Negative-stain transmission electron microscopy indicated both phages are members of *Siphoviridae* (Fig. 1).

**TABLE 1:** Assembly statistics for LilyPad and PokyPuppy genome assemblies

Phage	GPS coordinates	Genome length (bp)	Fold coverage (×)	GC content (%)	Character of ends^*a*^	Cluster	Subcluster
LilyPad	34.049565, −106.924937	64,158	1,160	59.2	Circularly permuted	DG	DG1
PokyPuppy	34.062750, −106.903575	77,065	836	59.1	Direct terminal repeats	CS	CS2

a Genome ends were determined as described previously (5).

DNA was isolated using the Wizard DNA cleanup kit (Promega, Madison, USA). DNA libraries were prepared using the New England BioLabs (NEB) Ultra II library kit and sequenced on an Illumina MiSeq (v3 reagents), using 150-bp single-end reads. This procedure yielded 523,290 reads for LilyPad and 452,482 reads for PokyPuppy. Software tools employed default parameters, except where noted. Genome sequences were assembled using Newbler v2.9 and Consed v29, as described previously (5). Sequencing results and genome characteristics are described in Table 1. The GC content of both phages was less than that of the host *G. rubripertincta* (67.5%). LilyPad and PokyPuppy were assigned to subclusters DG1 and CS2, respectively, based on gene content similarity (GCS) of 35% or higher to phages in the Actinobacteriophage database (https://phagesdb.org/), using the PhagesDB GCS tool (3, 6). GCS analyses indicate that LilyPad is the most distantly related phage among the phages in subcluster DG1.

Autoannotation was performed using GLIMMER v3.02b (7) and GeneMarkS v2.5p (8) embedded within DNA Master v5.23.6 (9), and start sites were manually refined using Phamerator (10), Starterator v474 (http://phages.wustl.edu/starterator/), and PECAAN v20211202 (https://discover.kbrinsgd.org/login). LilyPad encodes 91 genes, which are all transcribed on one strand, whereas PokyPuppy encodes 105 genes, which are transcribed on both strands, as well as one predicted tRNA (anticodon, GTT, asparagine) identified by Aragorn v1.2.41 (11) and tRNAscan-SE v2.0 (12).

Using BLASTP v2.2.26 (8) and HHPred v3.3.0 (13), 35 genes (~39%) in LilyPad and 29 genes (~27%) in PokyPuppy could be assigned the following putative functions. As with other subcluster CS2 phages, one-half of the LilyPad genome encodes structure and assembly functions, whereas the other half contains genes involved in DNA metabolism. The genes transcribed in one direction of the PokyPuppy genome (SEA_POKYPUPPY_8 through SEA_POKYPUPPY_38) include structure and assembly genes as well as two lysin A genes, whereas the genes transcribed on the other strand include various DNA metabolism genes and a lysin B gene.

### Data availability.

Assemblies are available at GenBank (accession no. ON970620 and ON456331). Reads have been deposited in SRA (accession no. SRX14483212 and SRX14483236).
